# SMS SOS: a randomized controlled trial to reduce self-harm and suicide attempts using SMS text messaging

**DOI:** 10.1186/s12888-019-2104-9

**Published:** 2019-04-18

**Authors:** Garry J. Stevens, Trent E. Hammond, Suzanne Brownhill, Manish Anand, Anabel de la Riva, Jean Hawkins, Tristan Chapman, Richard Baldacchino, Jo-Anne Micallef, Jagadeesh Andepalli, Anita Kotak, Naren Gunja, Andrew Page, Grahame Gould, Christopher J. Ryan, Ian M. Whyte, Gregory L. Carter, Alison Jones

**Affiliations:** 1 School of Social Sciences and Psychology, Western Sydney University (WSU), Kingswood, NSW Australia; 2 Triage and Assessment Centre, Mental Health Centre, Nepean Hospital, Nepean Blue Mountains LHD, Kingswood, NSW Australia; 3 Consultation Liaison Psychiatry, Westmead Hospital, Western Sydney Local Health District (WSLHD), Westmead, NSW Australia; 4 Consultation Liaison Psychiatry, Blacktown Hospital, WSLHD, Blacktown, NSW Australia; 5 Specialist Mental Health Older People Service, Mental Health Centre, Nepean Hospital, NBMLHD, Kingswood, NSW Australia; 6 Child and Youth Consultation Liaison, Nepean Hospital, NBMLHD, Kingswood, NSW Australia; 7 Psychiatry, Cumberland Hospital, WSLHD, Westmead, NSW Australia; 8 Department of Clinical Pharmacology and Toxicology, Western Sydney Health, Westmead, NSW Australia; 9 Translational Health Research Institute, School of Medicine, WSU, Campbelltown, NSW Australia; 10 Illawarra Institute for Mental Health, University of Wollongong, Wollongong, NSW Australia; 11 Sydney Medical School, University of Sydney, Sydney, NSW Australia; 12 Department of Clinical Toxicology and Pharmacology, Calvary Mater Hospital Newcastle, Waratah, NSW Australia; 13 School of Medicine and Public Health, Faculty of Health and Medicine, Newcastle University, Callaghan, NSW Australia; 14 Vice Chancellor’s Unit, Faculty of Science, Medicine and Health, University of Wollongong, Wollongong, NSW Australia

**Keywords:** Deliberate self-harm, Intentional self-harm, Prevention, Randomized controlled trial, Reattempt, Re-present, Short message service, SMS, Suicide, Text message

## Abstract

**Background:**

Hospital-treated deliberate self-harm (DSH) is common, costly and has high repetition rates. Since brief contact interventions (BCIs) may reduce the risk of DSH repetition, we aim to evaluate whether a SMS (Short Message Service) text message Intervention plus Treatment As Usual (TAU) compared to TAU alone will reduce hospital DSH re-presentation rates in Western Sydney public hospitals in Australia.

**Methods/design:**

Our study is a 24-month randomized controlled trial (RCT). Adult patients who present with DSH to hospital emergency, psychiatric, and mental health triage and assessment departments will be randomly assigned to an Intervention condition plus TAU receiving nine SMS text messages at 1, 2, 3, 4, 5, 6, 8, 10 and 12-months post-discharge. Each message will contain telephone numbers for two mental health crises support tele-services. Primary outcomes will be the difference in the number of DSH re-presentations, and the time to first re-presentation, within 12-months of discharge.

**Discussion:**

This study protocol describes the design and implementation of an RCT using SMS text messages, which aim to reduce hospital re-presentation rates for DSH. Positive study findings would support the translation of an SMS-aftercare protocol into mental health services at minimal expense.

**Trial registration and ethics approval:**

This trial has been registered with the Australian and New Zealand Clinical Trials Registry (Trial registration: ACTRN12617000607370. Registered 28 April 2017) and has been approved by two Local Health Districts (LHDs). Western Sydney LHD Human Research Ethics Committee approved the study for Westmead Hospital and Blacktown Hospital (Protocol: HREC/16/WMEAD/336). Nepean Blue Mountains LHD Research Governance Office approved the study for Nepean Hospital (SSA/16/Nepean/170).

## Background

Hospital-treated DSH is common [1] and costly [2]. Repetition of DSH within 1 year of hospital admission occurs at a median proportion of 15% (interquartile range 12–25%) [1].

DSH is associated with a range of psychiatric issues [3], an increased risk of suicide attempts, and suicide [3, 4]. In Australia, DSH accounted for 6% of all hospitalised injury cases during 2012–2013 and four-billion dollars in healthcare expenditure annually, making prevention and early intervention an Australian national health priority [5]. Hospital-treated DSH predominantly includes deliberate self-poisoning (DSP) (approximately 90% of DSH cases) and other various methods, including, cutting, hanging, jumping, and burning [1]. Hospital-treated DSH is associated with an increased risk of suicide death, highlighting the importance of effective prevention and early intervention strategies.

BCIs, such as follow-up postcards, supportive letters, or phone calls have previously been shown to be associated with reductions in hospital re-presentation event rates. A recent systematic review and meta-analysis of BCIs for reducing DSH (letters, telephone calls and crisis “green cards”) found a non-significant benefit for any episode of repeat DSH (binary outcome) and a significant benefit for repetition event rates [6]. The individual studies identified in the systematic review and meta-analysis generally lacked statistical power and varied considerably in intervention timeframes and methodologies, highlighting the need for well-designed, large, RCTs to determine the efficacy of these BCIs.

SMS text messaging provides more immediate communication and may be a more effective intervention than postcards [6, 7]. Text messaging has been associated with positive outcomes for hospital patients in a pilot study [8] and in a prospective study [9]. These studies demonstrated the technical feasibility and acceptability of text messaging outreach in post-acute suicide attempters in different cultural contexts. Most participants considered the text message contacts an acceptable and useful form of help and indicated they wished to continue receiving those text messages.

If an SMS text messaging follow-up intervention is found to be effective in reducing DSH re-presentations, it would provide a lower-cost, higher coverage medical follow-up system than what has previously existed for this population.

## Methods/design

### Aims and hypotheses

The aim of this study is to investigate whether Treatment As Usual (TAU) aftercare for DSH patients plus supportive SMS text messages delivered over 1 year reduce DSH re-presentations to hospital, compared to TAU alone. Our hypotheses are: 1) SMS text messages *plus* TAU will be associated with a significantly lower incidence event rate of hospital DSH re-presentations, compared to the patient group receiving TAU; and 2) SMS text messages *plus* TAU will be associated with a significantly greater time to first hospital DSH re-presentation compared to TAU.

### Study design

A RCT will be conducted in three selected public hospitals in Western Sydney, Australia, and will have a 24-month recruitment period and a 12-month follow-up period. The study protocol is like the methodology employed in an earlier study [10], which was a two-year French multicenter RCT, comprising of DSH patients, similar text messages, and an SMS outreach schedule of 48 h, 7 days, 15 days and, then monthly for 12-months. Our study consists of a parallel design that will compare a control group (TAU condition) with patients who receive an Intervention condition (i.e. SMS text messages *plus* TAU). Outcome data on DSH will be obtained from routinely collected hospital records, extracted by trained research staff.

### Setting

Our study will be conducted at three large, public hospitals in Western Sydney; Nepean, Blacktown and Westmead Hospitals.

### Participants/Subjects

For the purpose of this study, participants will be defined as patients who have randomly been allocated to the Intervention condition and provide their informed consent to receive text messages. Subjects will be defined as patients who are randomly allocated to the TAU condition and do not receive text messages. To be eligible for study inclusion, patients must be: assessed at an emergency department (ED) after presenting with DSH; at least 16-years old; competent in reading and understanding English; and have a mobile phone. Patients will be excluded from the study if they: refuse to participate; do not have a mobile phone; do not have a fixed Australian address or are otherwise unable to provide informed consent.

### Randomization, allocation, and materials

Participants/subjects will be stratified (first ever hospital-treated DSH verses any subsequent episode), then randomly assigned and enrolled into the study following the Zelen single-consent design [11]. Since there are different repetition rates of DSH for initial and subsequent DSH, participant/subject numbers will initially be stratified by first or subsequent DSH presentation. They will then be randomized within strata, using random permuted blocks of size 6, to either the Intervention or control condition (TAU) using a computer-generated randomization program.

Study materials, including an ‘Allocation Information Sheet,’ will be organized within numbered envelopes, with each hospital having at least two ‘enrolment boxes’ (document drawers) containing a full set of materials. Research staff will confirm that the placement of research materials and the Allocation Information Sheets within envelopes match the randomized numbers generated from the randomization program (to prevent any tampering with the allocation sequence).

### Data collection

Clinical and demographic information of patients will be recorded, including: presenting group (i.e. first DSH or subsequent DSH presentation) randomized condition (i.e. TAU or Intervention); mobile phone number; hospital of study enrollment; key dates (admission, enrollment, discharge, and deceased dates); clinical diagnoses; primary DSH method; gender, birth date, place of residence, marital status, primary language spoken, Australian Aboriginality status, and ethnicity), Medical Record Number, first name, and last name.

### Procedure

The usual treating health and mental health clinicians (Psychiatrists, Clinical Nurse Consultants, Registered Nurses and Psychiatry Registrars) will assess patients at EDs, Toxicology Centers, Psychiatric Emergency Care Centers, and Mental Health Triage and Assessment Centers. Clinicians will enroll eligible patients into the Intervention or TAU conditions and record the name, Medical Record Number, mobile phone number and date of birth of participants/subjects on Allocation Information Sheets. Envelopes will be supplied at study commencement, including at least 500 green envelopes (numbered for first DSH presentation) and at least 500 blue envelopes (numbered for subsequent DSH presentation). Allocation Information Sheets will be concealed inside sealed envelopes and the allocating clinician will draw the next numbered envelope for each participant/subject form the appropriate stratification. Demographic information will be collected by hospital administration staff when patients first present to the ED.

**Fig. 1:**
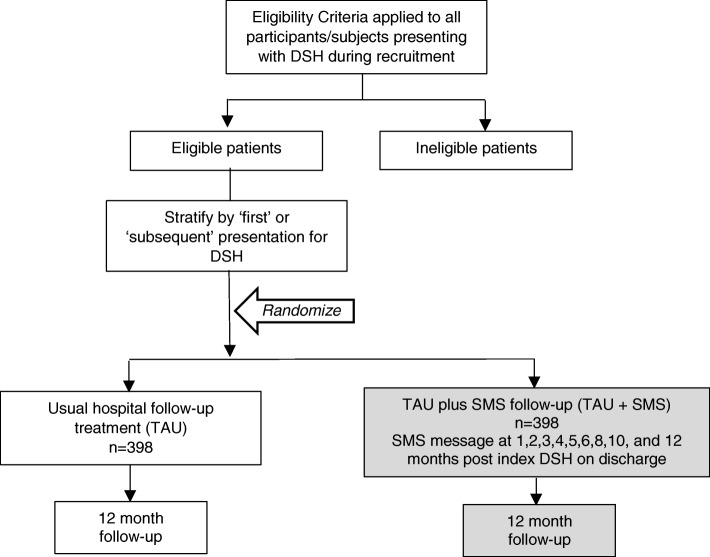
RCT design flowchart

Figure 1 outlines the methodological process that will be followed for the SMS SOS Study. Allocation will occur via numbered sealed envelopes. Upon enrolling patients into the study, health professionals will attach a patient identification label (which includes the patient’s name, gender, date of birth and Medical Record Number) to an Allocation Information Sheet, which would be drawn from a selected envelope. For patients allocated to the Intervention condition, clinicians will explain that they may receive text messages for up to 12-months post discharge. Clinicians will provide patients in the Intervention condition with a Participant Information Sheet and they will obtain written consent. To reduce unnecessary patient burden, patients allocated to the control condition (TAU) will not be recruited by clinicians, as per the Zelen single-consent design [11].

**Fig. 2:**
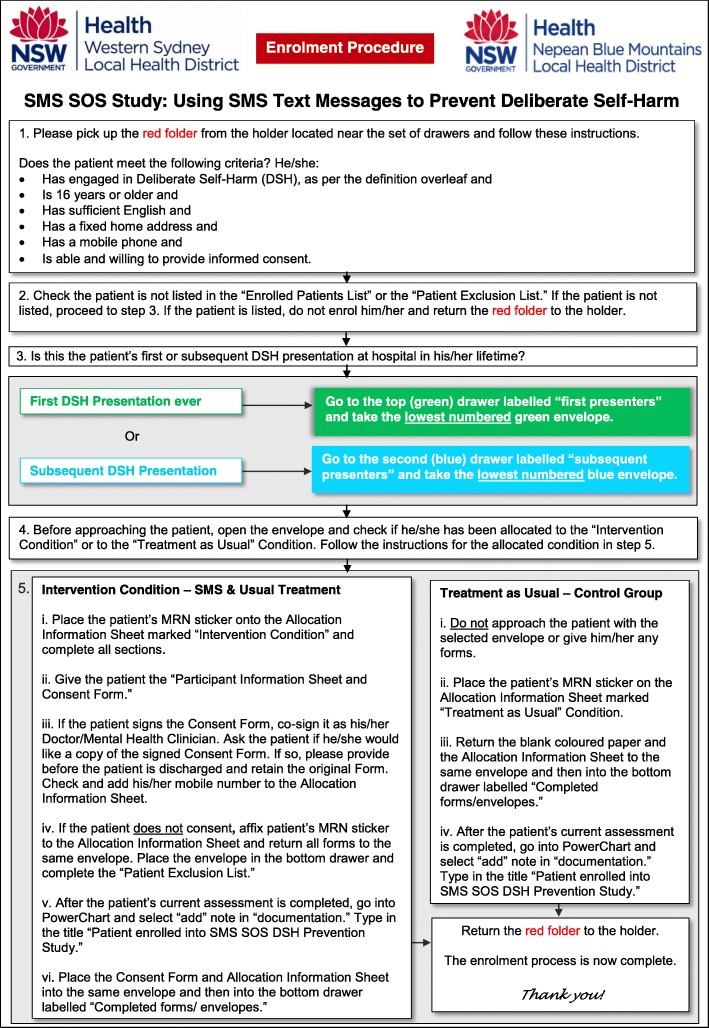
Enrolment procedure flowchart

To reduce the chance of patients being re-enrolled into the study, treating clinicians will review an Enrolled Patients List and a Patient Exclusion List placed in a folder above each enrollment box to be checked before recruitment. A hardcopy of the Enrolled Patients List and the Patient Exclusion List will be used due to the limited capacity of the patient electronic medical record system to reliably identify (‘flag’) to clinicians those patients who have previously been enrolled into the study. The folder will also contain an Enrollment Procedure Flowchart (Fig. 2) regarding the enrollment process and the definition of DSH used for the study.

### Intervention

Patients in the Intervention condition will receive TAU *plus* a series of supportive SMS text messages. The Intervention condition will consist of nine text messages in total, which were developed based on feedback received from a panel of lived experience mental health consultants. Seven people with mental health problems and/or a history of DSH informed the development of the text messages. According to the feedback received, each text message was clear and easy to read, and suited the purpose of making brief contact and offering support phone numbers.

The text messages will be personalized with the names of patients and will be sent with identical outreach schedules. Nine automated text messages will be sent to patients assigned to the Intervention condition at months 1, 2, 3, 4, 5, 6, 8, 10, and 12 post-discharge. This is like the contact schedule employed in the Postcards from the Edge study [12]. There will be three different SMS text messages sent to participants, which express concern for their wellbeing and encourage them to telephone clinical crises and mental health support services, including Lifeline and NSW Health’s ‘Mental Health Line,’ if needed. Patients in the Intervention condition will be advised once in the first text message that they will not be able to directly reply to text messages. The three text messages sent to patients in the Intervention condition are as follows:

Text message 1.

*Dear [name].*

*We hope that things have been going well for you since we last had contact.*

*Just a reminder that the 24-h contact line (13 11 14) is there if you’d like to connect with someone and Helpline staff (1800 011511) can put you in touch with your local health service if needed.*

*Best wishes. [Return SMS messages are unavailable from this service.]*

Text message 2.

*Hi [name].*

*We hope that you’ve been ok since our last contact. We’re just checking in with you.*

*A 24-h phone line is there for you in case you’d like to connect with someone (13 11 14) or to contact your local health service (1800 011511).*

*Best wishes.*

Text message 3.

*Dear [name].*

*Just checking in with you.*

*A reminder that help is there if you need it. Just call (13 11 14) or (1800 011511) for support.*

*Best wishes.*

The SMS text messages will be scheduled for automatic distribution via a private and as secure as possible medium, considering available Australian telecommunications providers. The telecommunications provider was selected based on its capacity to provide both an automated service with message variants and to personalize messages with the names of specific recipients. These features will support large scale, low cost delivery of a future mental health aftercare service for this population. The three SMS text messages will be delivered to participants according to the schedule detailed in Table 1.

**Table 1:** Delivery schedule for the three supportive SMS text messages

Text message	Month/s to be delivered
1	1 only
2	2, 4, 6 and 10
3	3, 5, 8 and 12

### Treatment as usual (control) condition

Patients assigned to the control condition will receive TAU without SMS text messages. This will vary for each patient but usually consists of hospital and/or community mental health contact with the presenting individual, typically within 24-h of their hospital presentation, and a follow-up program of care including clinical reassessment, appropriate mental health services, drug and alcohol services and/or general practitioner follow-up and indicated psychological therapies.

### Outcome measures

As primary endpoints, we will assess at 6 and 12-months post-index discharge, i) the number of hospital DSH re-presentations in each condition (repetition event rate) and ii) the time to first re-presentation in each condition. Secondary end-points include i) any DSH re-presentation, ii) suicide mortality, and iii) all-cause mortality. Mortality data will be obtained from routinely collected data sources available through NSW Health, other government agencies, and the Centre for Health Record Linkage (CheReL).

### Sample size calculation

Assuming an incidence rate ratio (based on event rates) of RR = 0.66 [13] for the Intervention condition compared to TAU, with a 5% significance level, 80% power, and 10% adjustment for correlation of individuals within hospitals, a total sample size of 796 participants/subjects is required (398 participants in the Intervention condition and 398 subjects in the control condition). We have also assumed a median survival time (time to first repetition) in the TAU condition of 4.3 years. Currently ~ 15% in the usual care group who present for DSH re-present within the following 12-months, or a probability of survival of 0.85. Median survival (m) after 1-year (t) in the usual care group is t*log_e_(1/2)log_e_(*p* = 4.3 years). For assessing differences in median time to first repetition, assuming a median survival time in the usual care group of 73.5 days [12] and a similar relative difference in event rates between Intervention and TAU conditions as above (RR = 0.66), [13] the estimated median survival time in the Intervention condition is 121.8 days, which requires a total sample size of 138 participants/subjects (69 participants in the Intervention condition and 69 subjects in the TAU condition), with 5% significance and 80% power, and 10% adjustment for correlation of individuals within hospitals. The number of patients likely to die within the study period is very small. A previous longitudinal study in a similar Australian hospital setting [12] found a 1% suicide rate after 24 months and nearly a 2% suicide rate after 5 years.

### Statistical methods

Participant/subject characteristics will be summarized using mean, standard deviation, median and inter-quartile range. Categorical variables will be summarized using frequencies, percentages and 95% confidence intervals. Means will be compared between the Intervention condition and TAU condition using the t-test or Mann-Whitney test. Comparisons of proportions between conditions will be performed using the Chi square test or Fisher’s exact test, as appropriate. Differences in the incidence of subsequent DSH between the Intervention and TAU conditions at 6 and 12 months will be investigated using multi-level negative binomial regression models (individuals nested within hospitals) to estimate the incidence risk ratios (IRR) and absolute risk differences based on incidence proportions in an intention to treat analysis. Adjustment for potential confounders identified from descriptive analyses will also be included in multivariate regression models as appropriate.

Relative differences in time to first subsequent DSH event between Intervention and control conditions at 6 and 12 months will also be investigated using multi-level Cox proportional hazard regression models (individuals nested within hospitals) to estimate hazard ratios (HR), with adjustment for potential confounders as appropriate. Kaplan-Meier survivorship functions and clustered log-rank tests will also be calculated to assess differences between Intervention and TAU conditions, with median time to first DSH event compared for Intervention and TAU conditions.

### Ethical considerations

Participants in the Intervention condition will be informed that they can withdraw from the study at any time by contacting the relevant study coordinator at the hospital of original enrolment. The study has been approved by the NSW Ministry of Health and the Western Sydney Local Health District Human Research Ethics Committee (Protocol Number: HREC/16/WMEAD/336). Site specific approval was granted by the Nepean Blue Mountains Local Health District Research Governance Office (SSA/16/Nepean/170).

## Discussion

The present article details the research protocol for an RCT to investigate the impacts of SMS aftercare on DSH hospital re-presentations. BCIs have been found to be effective at reducing DSH re-presentation rates. SMS aftercare offers a more immediate contact medium, which is increasingly relevant across all demographic groups, but especially among young people; a key risk group for DSH and suicide. SMS aftercare also has the potential to augment existing mental health programs and provides a ready platform for the provision of psychosocial information and interactive tools. Should SMS aftercare prove effective in reducing DSH re-presentations, it would allow for a significantly more flexible, low-cost and deliverable medical follow-up system than what currently exists.

## Abbreviations

BCI: Brief contact intervention
DSH: Deliberate self-harm
DSP: Deliberate self-poisoning
RCT: Randomized controlled trial
SMS: Short Message Service
TAU: Treatment As Usual
